# Investigating the Relationship Among English Language Learning Strategies, Language Achievement, and Attitude

**DOI:** 10.3389/fpsyg.2022.867714

**Published:** 2022-05-13

**Authors:** Anita Habók, Andrea Magyar, Gyöngyvér Molnár

**Affiliations:** ^1^Institute of Education, University of Szeged, Szeged, Hungary; ^2^MTA–SZTE Digital Learning Technologies Research Group, Szeged, Hungary

**Keywords:** self-regulated foreign language learning, language learning strategies, foreign language attitude, language achievement, lower secondary students

## Abstract

The main objective of the study was to ascertain whether English as a Foreign Language learners with various levels of English language achievement differ significantly in applying foreign language learning strategies. We also aimed to explore strategy use frequency in connection with attitude toward English language learning. Data were collected from 1,653 lower secondary students in Hungary through a revised version of the previously developed online Self-Regulated Foreign Language Learning Strategy Questionnaire (SRFLLSQ) based on Oxford’s Strategic Self-Regulation (S^2^R) Model. The findings point to statistically significant differences in the frequency of English language strategy use among more and less proficient learners. Quantitative analyses also reported that, in spite of the students stated low or moderate levels of strategy use, it turned out as a statistically significant predictor of foreign language attitude and language achievement. The results draw attention to the relevance of strategy research in foreign language teaching as well as encourages strategy teaching within language instruction.

## Introduction

Foreign language learning requires many underlying skills and techniques. Learners have to master a number of complex linguistic, personal, cultural and social skills, and competences and be aware of effective techniques and strategies to help them cope with various challenges during the learning process. The frequent use of learning strategies can help learners to become more competent and effective language users in the classroom and inspire them to achieve higher levels of mastery in the target foreign language (Wong and Nunan, 2011; Oxford, 2016). Since the mid-1970s, an immense amount of learning strategy research has attempted to establish the concept and identify strategies that help learners to become more effective language learners (Oxford, 1990; Cohen, 1998). It is a widely researched and highly debated area even today (Thomas and Rose, 2019; Thomas et al., 2021). The most well-known and widely used taxonomy of language learning strategies (LLS) was developed by Oxford (1990, 2011, 2016). In her recently reconsidered Strategic Self-Regulation (S^2^R) Model based on Vygotsky’s (1978) sociocultural theory of self-regulated learning (SRL) and Zimmerman’s three-phase model (Zimmerman, 2000; Zimmerman and Schunk, 2011), she identified four main strategy categories: cognitive, affective, motivational, and social, each of them guided by the master category of “meta-strategies.” These meta-strategies are metacognitive, meta-affective, metamotivational, and metasocial strategies, respectively (Oxford, 2016).

Oxford also developed a measurement tool (Strategy Inventory for Language Learning, SILL) for investigating LLS use, which is employed worldwide; however, it is based on her original conceptualization. Nevertheless, it is essential to relate the latest pedagogical theories to language teaching practice. Self-regulation theory, which was the basis for Oxford’s new taxonomy, has been dominant since the beginning of this century. It is thus crucial to develop state-of-the-art measurement tools which can be used in the classroom by language teachers. In previous research, we have developed and validated a questionnaire based on Oxford’s S^2^R Model (SRFLLSQ; Habók and Magyar, 2018b). To obtain a more comprehensive view of the role and possible effect of language learning strategies on certain other factors, such as attitude, motivation, and self-efficacy, it is essential to conduct further research. In this study, we aimed to examine LLS in relation to other crucial factors of language learning; we have investigated the relationships among the application of English language learning strategies, language achievement, and attitude toward English among lower secondary students in Hungary.

## Literature Review

### The Concept of Language Learning Strategies

Language learning strategies have been a research focus since the mid-1970s, as strategic language learning is a key to successfully acquiring a foreign language (Rubin, 1975). A number of definitions of LLS have emerged, with one of the most influential having proved to be that of Rebecca Oxford, who not only established a conceptualization, but also conducted empirical research. In her content-analytic study, Oxford involved 33 distinct definitions and interpretations of the term LLS and thus determine it as follows:

L2 learning strategies are complex, dynamic thoughts, and actions, selected and used by learners with some degree of consciousness in specific contexts in order to regulate multiple aspects of themselves (such as cognitive, emotional, and social) for the purpose of (a) accomplishing language tasks; (b) improving language performance or use; and/or (c) enhancing long-term proficiency. Strategies are mentally guided but may also have physical and therefore observable manifestations. Learners often use strategies flexibly and creatively; combine them in various ways, such as strategy clusters or strategy chains; and orchestrate them to meet learning needs. Strategies are teachable. Learners in their contexts decide which strategies to use. Appropriateness of strategies depends on multiple personal and contextual factors (Oxford, 2016, p. 48).

Strategic language learners select their LLS according to their personal preferences, motivational intentions, and situational circumstances. Therefore, it is especially difficult to identify a system for these strategies. This is one of the reasons why an extremely large number of conceptualizations and debates have emerged (Thomas and Rose, 2019; Thomas et al., 2021). Thomas et al. (2021) have pointed out that with the emphasis on self-regulation, the field of strategy research has moved away from formal educational settings toward learner autonomy. They argue that this is a hazardous trend because definitions of LLS minimize teachers’ role and classroom contexts that can also be an influencing factor in strategic behavior among students. Thomas and Rose (2019) have therefore suggested a separation of LLS from self-regulation and introduced the Regulated Language Learning Strategies Continuum to make it clear that self-regulation can be conceptually separated in defining LLS. By interpreting LLS as being both other- and self-regulated, Dörnyei and Skehan (2003) argue that strategy use cannot be regarded as either emotional or cognitive or even behavioral, thus opening up another debated area in the field.

The classification of LLS is another key area of argument. Oxford’s original classification of six major fields (memory, cognitive, metacognitive, affective, compensation, and social strategies) was recently reconsidered and restructured on the basis of self-regulation theories. Her Strategic Self-Regulation (S^2^R) Model was developed based on Vygotsky’s (1978) sociocultural theory of self-regulated learning (SRL). In her concept, she identified four main fields—cognitive, affective, motivational, and social strategies—each of them directed by a “master category of meta-strategies.” These meta-strategies are metacognitive, meta-affective, metamotivational, and metasocial strategies (Oxford, 2016). Her taxonomy was again open to a number of debates as some theorists (Dörnyei, 2005; Hajar, 2019) argued that success in language learning cannot be assessed through the frequency of strategy use alone.

### Research on Language Learning Strategies

Despite the debates, LLS researchers have been devoted to conducting quantitative research on LLS use and its connection with other individual factors, such as gender, learning style, motivation, attitude, and proficiency (e.g., Radwan, 2011; Alhaysony, 2017; Habók and Magyar, 2018a, 2019). The most widespread measurement tool for assessing L2 learners’ strategy use is Oxford’s Strategy Inventory for Language Learning (SILL; Oxford, 1990). This questionnaire has been translated into numerous languages and adapted for a vast number of cultures around the world. It is based on her original taxonomy and employs her original six strategy fields. Based on her renewed taxonomy, a number of reconsidered measurement tools have been developed since then, which have approached effective language learning from different perspectives (Wang et al., 2013; Salehi and Jafari, 2015; Božinović and Sindik, 2017; Köksal and Dündar, 2017; Habók and Magyar, 2018b; An et al., 2021).

One major area for researchers has been to find out what set of strategies foreign language learners rely on the most (Platsidou and Sipitanou, 2015; Alhaysony, 2017; Charoento, 2017; Dawadi, 2017; Habók and Magyar, 2018a,b, 2019, 2020; Habók et al., 2021). Overall, results have concluded moderate use of LLS among participants. The most frequently used strategies are cognitive, metacognitive, and compensation strategies, while affective and memory strategies are the least preferred. Habók et al. (2021) have pointed out the different strategy preferences in different cultural contexts. Their results reinforced the preferred use of metacognitive strategies in both European and Asian contexts. However, there were statistically significant differences in the affective field with regard to the lower strategy use preference in the European sample.

A great deal of research has investigated strategy use in connection with other aspects (Platsidou and Kantaridou, 2014; Rao, 2016; Charoento, 2017; Habók and Magyar, 2018a, 2020). One of the most often used factors was language achievement, which has been specified and covered in a multitude of ways. Some studies have focused on level of language proficiency or achievement test results (Raoofi et al., 2017; Taheri et al., 2019; An et al., 2021; Malpartida, 2021), others have relied on self-ratings (Charoento, 2017), and still others have involved language course marks (Habók and Magyar, 2018a; Sánchez, 2019; Bećirović et al., 2021). As a result, most research has pointed out that students with higher proficiency use LLS more frequently than those with less (Rao, 2016; Charoento, 2017; Raoofi et al., 2017; Sánchez, 2019). Charoento (2017) highlighted that successful students mainly used metacognitive strategies and less proficient students preferred to use social strategies the most. Sánchez (2019) reported that the application of social, metacognitive, and cognitive strategies was the highest among high achievers. Some research failed to find any significant differences in LLS use between learners with lower and higher English proficiency levels (Rianto, 2020).

A relatively small number of studies have examined how LLS use predicts language proficiency. Some research has pointed out a positive correlation between strategy use and proficiency. Comprehensive work by Taheri et al. (2019) indicated a statistically significant correlation between LLS and second language achievement. Specifically, they confirmed a statistically significant relationship between cognitive, social, and compensation strategies and second language achievement. Platsidou and Kantaridou (2014) also found that language use is predicted by learning strategy use in a statistically significantly way and that it anticipates perceived language performance. Rao (2016) also reinforced that learners’ English proficiency level determines their strategy use and highlighted that students with high proficiency use strategies more frequently than low-level learners. Habók and Magyar (2018a) stated that strategies have a statistically significant effect on proficiency through attitudes. In addition, these effects reflect general school achievement. Bećirović et al. (2021) observed that LLS can influence students’ English as a foreign language (EFL) achievement. Specifically, cognitive strategies have a statistically significant positive effect on EFL achievement, while other strategies showed negative or no significant impact. An et al. (2021) also reported the positive direct effect of SRL strategies on students’ English learning outcomes. Lin et al. (2021) concluded the direct impact of learning strategies on learners’ performance in literal and inferential comprehension.

Another research direction is the investigation of strategy use in relation to other underlying factors, like affective variables, such as motivation, attitude, self-efficacy, and self-concept. Educational research has pointed out that learners’ attitude toward language learning is crucial since it can greatly impact learning results and language learning proficiency (Platsidou and Kantaridou, 2014). Studies have indicated that learners with a positive attitude toward language learning employ LLS more frequently and effectively. Platsidou and Kantaridou (2014) used confirmatory factor analysis to show that attitudes toward language learning predict the use of both direct and indirect learning strategies. Jabbari and Golkar (2014) reported a more frequent use of cognitive, metacognitive, compensation, and social strategies among students with a positive attitude toward language learning. Habók and Magyar (2018a) demonstrated the reverse effect: learners who apply LLS effectively were more likely to have improved learning experiences and positive attitudes toward foreign language learning.

It can be concluded that strategy research is often related to other vital areas of language learning, among which attitude plays an important role. However, only a limited number of researchers have developed measurement tools for investigating self-regulated foreign LLS and measured it in relation to attitude. In addition, most studies have focused on the strategy use of tertiary samples with relatively high levels of proficiency. This study aims to fill this gap and provides an insightful investigation into the connections among strategy use, attitude, and English language achievement among lower secondary students. Based on the relevant literature (Jabbari and Golkar, 2014; Platsidou and Kantaridou, 2014; Habók and Magyar, 2018a), we hypothesized the statistically significant effect of LLS on proficiency through attitude.

## Research Questions

The research addresses the following three research questions:

1. Which EFL strategy was the most frequently used by 11-year-old lower secondary students?
2. Were there statistically significant differences among students in their language learning strategy use on the basis of their English language achievement?
3. Which language learning strategy type has a statistically significant impact on learners’ English language achievement and attitude?

## Research Methods

### Participants

In Hungary, students start primary school at the age of six. This lasts 4 year. Then, they continue their studies at the lower secondary level. At the age of 14, they move on to upper secondary school. The participants of the present research were 11-year-old lower secondary students in Grade 5 (N_total_ = 1,653; N_boys_ = 827, N_girls_ = 780, N_missing_ = 46) from 64 schools in Hungary. Hungarian students take EFL in compulsory courses in school, and they usually start learning a foreign language at the age of nine. However, in some schools, they can start at the age of six. Typically, they can choose between English and German, but recently a preference for English has become more common. In upper secondary school, two foreign languages are compulsory, English and German or Italian or Spanish. The second language depends on curricular choice at the school level.

The English proficiency of the participating students was at beginner/elementary level (A1–A2). As for their engagement in learning, there were 17 students in the sample who spent 2 h or less per week on English. Around half of the learners (*N* = 884) devoted 3 h a week to this subject, and few participants dedicated four (*N* = 303) or five (*N* = 357) hours a week to the language. We also found 67 students who dealt with English six or more hours per week. In addition, we did not receive any answers to this question from 25 students.

### Instrument

The revised and improved version of the Self-Regulated Foreign Language Learning Strategy Questionnaire (SRFLLSQ) was employed after our first measurement and validation (Habók and Magyar, 2018b). We reviewed the new findings on the theoretical background to foreign LLS research and continued revising the affective field. In addition, based on the relevant literature, we included the field of motivation in the questionnaire. We thus completed the measurement tool with motivational and metamotivational factors based on Oxford’s Strategic S^2^R Model. Finally, the questionnaire covered four strategy areas: metacognitive (eight items), cognitive (six items), meta-affective (eight items), affective (eight items), metasocial (eight items), social (six items), motivational (four items), and metamotivational (four items; see Appendix). The fifth-grade students provided their responses on a five-point Likert scale, which ranged from 1 (“Never or almost never true of me”) to 5 (“Always or almost always true of me”). The measurement tool was also complemented with a background questionnaire, which asked students about their foreign language school marks, which indicated students’ English language achievement (1 = fail, lowest school mark; 5 = excellent, highest school mark). Students also self-reported their attitudes toward English learning on a five-point Likert scale, which again ranged from 1 to 5.

### Procedure

First, the research was accepted by the IRB at the University of Szeged Doctoral School of Education. It was concluded that the research design follows IRB recommendations. The participating learners’ parents were asked for written informed consent, which was handled by the participating schools. Second, an invitation was sent to schools to register for the measurement. In the call, schools were given information about the purpose of the measurement. Once the schools accepted the invitation, they received further instructions on data collection and a link to log into the Online Diagnostic Assessment System (eDia), which is developed, supervised, and operated by the University of Szeged Centre for Research on Learning and Instruction (Csapó and Molnár, 2019). Students’ participation was voluntary in the research. They logged into the system with an official student assessment code (developed by the Hungarian Educational Authorities), which provided complete anonymity for them. The researchers could not identify the respondents on this basis. The identification code was handled by the school administrators, but the students’ results were not available to them. Thus, complete anonymity was guaranteed. The eDia system is familiar to students because they regularly use it for diagnostic purposes during official school hours. The students had already acquired the necessary ICT skills at primary level, further improved through remote learning. For the present questionnaire, the participants indicated their responses by clicking on radio buttons. The learners were given a school lesson in a personal classroom environment provided by the school. After logging in, the respondents filled in the questionnaire in Hungarian, which is their native language, because they do not yet have the foreign language skills to provide reliable answers in English. This took 20 min to complete the instrument. Teacher assistance was not required while the questionnaire was being completed, but it was available. The students had the option to ask for assistance on any technical problems.

### Data Analysis

First, we employed classical test analysis and examined reliability, means, and standard deviations for the questionnaire fields with SPSS Statistics 23.0. In the case of frequency of strategy use, we aimed to find out how strategy use was perceived by our sample. We also compared the students’ strategy use vis-à-vis their English language achievement and attitude using an independent sample *t*-test. To interpret effect size, we followed Wei et al.’s (2019) and Wei and Hu’s (2019) benchmark: under 0.005 is small, 0.01 is typical or medium, 0.02 is large, and is 0.09 very large. We used *R*^2^ unsquared; thus, the benchmark for the effect size index is 0.07, 0.10, 0.14, and 0.30, which, respectively, represents small, medium, large, and very large cut-off values. We applied path analysis to map the possible relationships and effects of our variables. We studied the goodness-of-fit indices by applying various cut-off values for many fit indices, including the Tucker–Lewis index (TLI), the normed fit index (NFI), the comparative fit index (CFI), the root mean square error of approximation (RMSEA), and Chi-square values (Kline, 2015). TLI, NFI, and CFI were regarded as eligible with a cut-off value of 0.95, and RMSEA values indicated an acceptable fit of 0.8 (Kline, 2015).

## Results

### Descriptive Analysis

The questionnaire was reliable in all the fields (Table 1). As regards the whole sample, moderate strategy use was observed. The lowest strategy use was noted in the field of metasocial strategies, and the most frequent strategy was found in the affective field. As regards the corresponding factors, the most frequent use was observed in the motivational field (see Table 1).

**Figure 1:**
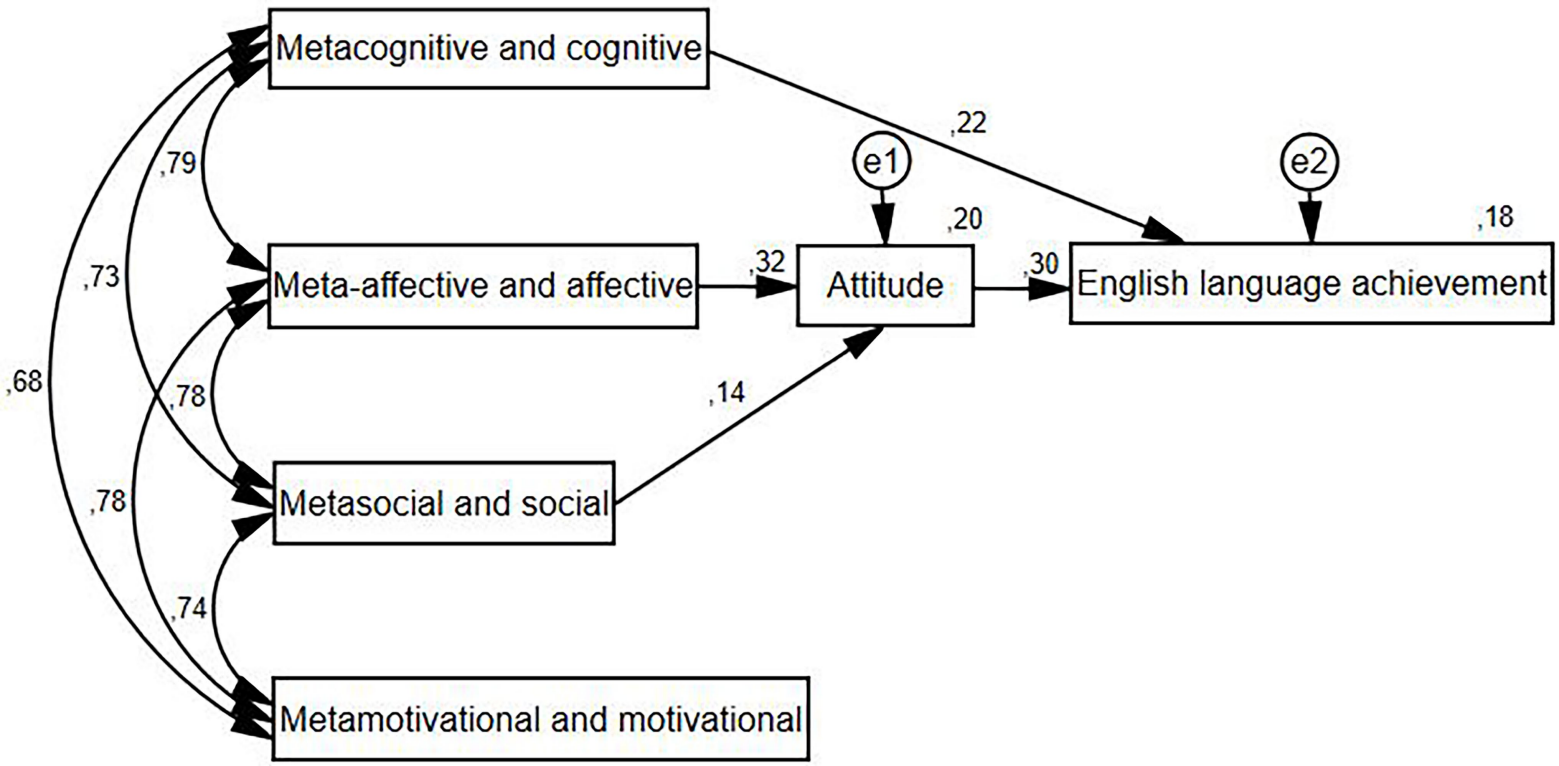
The path model for EFL strategies on English language achievement through attitude.

**Table 1:** Frequency of language learning strategy use in Grade 5.

Fields	Crb alpha	*M* (SD)	*M* (SD)
Metacognitive	0.79	3.47(0.74)	3.46(0.70)
Cognitive	0.72	3.43(0.78)	
Meta-affective	0.73	3.28(0.77)	3.55(0.72)
Affective	0.83	3.82(0.81)	
Metasocial	0.88	3.19(0.98)	3.28(0.92)
Social	0.85	3.41(0.94)	
Metamotivational	0.76	3.45(0.98)	3.60(0.84)
Motivational	0.66	3.75(0.90)	

We also identified the frequency of strategy use among the more and less proficient learners. Students were divided into two categories based on their English language achievement (Table 2). Those with excellent and good school marks were placed in the more proficient learners’ category, while learners with average, fair, or unsatisfactory school marks were grouped into the less proficient learner category. Students (*N* = 810) who received excellent school marks met the requirements of the English language curriculum and performed at a high level. Learners (*N* = 500) with good marks had minor gaps. Those (*N* = 229) with an average school mark had major gaps in their knowledge, and those (*N* = 65) with unsatisfactory school marks had difficulty following the curriculum and varying levels of difficulty in all areas of language learning. Finally, students (*N* = 9) who received an unsatisfactory school mark are at a disadvantage which is difficult to overcome. No data were received from 40 students. Students’ EFL achievement could be regarded as good with a mean of 4.2 (SD = 0.89). As a result, the more proficient learners employed strategies with greater frequency in all of the fields, a statistically significant finding. The affective factor indicated above medium effect size, while the other factors reported small effect sizes.

**Table 2:** Frequency of language learning strategy use among less and more proficient learners.

Fields	Less proficient learners *M* (SD)	More proficient learners *M* (SD)	*t*	*r* (effect size)
Metacognitive	3.02(0.71)	3.58(0.71)	−12.21^*^	0.085
Cognitive	3.07(0.74)	3.51(0.76)	−9.28^*^	0.050
Meta-affective	3.07(0.77)	3.33(0.76)	−5.24^*^	0.017
Affective	3.26(0.79)	3.96(0.76)	−13.85^*^	0.113
Metasocial	2.82(0.88)	3.27(0.98)	−7.30^*^	0.032
Social	2.98(0.86)	3.50(0.93)	−8.78^*^	0.046
Metamotivational	3.14(0.96)	3.51(0.98)	−5.91^*^	0.021
Motivational	3.32(0.97)	3.84(0.86)	−9.34^*^	0.052

* Differences are statistically significant at *p* < 0.001 level.

### Multivariate Analyses

Finally, we investigated the effect of strategy use on English language achievement and attitudes. As Oxford’s Strategic S^2^R Model considers strategies as being closely directed by their corresponding meta-strategies, we have regarded the strategies and their meta-strategy counterparts as common factors. The correlation coefficient was statistically significant between every strategy factor (*r* = 0.45–0.25, *p* < 0.001). Our model showed acceptable fit indices (Chi-square = 35.574, df = 5, *p* = 0.000, CFI = 0.995, TLI = 0.977, NFI = 0.994, RMSEA = 0.061). We therefore concluded that English language achievement and attitude are statistically influenced by strategy use (Figure 1).

We found that the meta-affective and affective and metasocial and social categories directly influenced students’ attitude toward English. A direct effect of attitude was observed on English language achievement. In addition, the metacognitive and cognitive categories had a direct effect on English language achievement, while English language achievement was indirectly affected by meta-affective and affective and metasocial and social factors. We could not confirm any significant effect of metamotivational and motivational factors on attitude or English language achievement.

## Discussion

We investigated the strategy use of 11-year-old lower secondary students in Grade 5 in connection with their language achievement and attitude toward the English language. Our first research question asked which LLS was the most frequently used by the sample. We found moderate strategy use with regard to a slightly modest application of the metasocial field, and the most frequent strategy use was observed in the affective field. These aspects of our findings partly correspond with most of the recent research with respect to moderate use of strategies; however, there are profound differences in the strategy preferences of the sample (Platsidou and Sipitanou, 2015; Alhaysony, 2017; Charoento, 2017; Dawadi, 2017; Habók and Magyar, 2018a,b, 2019, 2020; Habók et al., 2021). Raoofi et al. (2017) also pointed out the low level of social strategy use in their research. Another important statistically significant finding is that higher proficiency learners used learning strategies with greater frequency than their less proficient peers. This applies to every strategy field in agreement with Charoento’s (2017) results.

Our second research question concerned differences in the use of LLS based on English language achievement. As concerns the sample, we regarded the EFL school mark as an indicator of English language achievement. The mean indicated that a considerable portion of the sample was grouped as more proficient. As a result, these students used LLS with greater frequency in all of the categories, which is a statistically significant finding. These results correspond with other research, which also reinforces this (Rao, 2016; Charoento, 2017; Raoofi et al., 2017; Sánchez, 2019). However, we also found that less proficient learners employed motivational strategies the most frequently, while their more proficient peers most often preferred the affective field, a result which is not reinforced by any previous findings. Apart from this, the strategy uses of both subsamples followed the same order, with social and metasocial strategy use being the least preferred type for both. This may be due to the fact that our sample was mainly at the beginner/elementary level (A1–A2), so they cannot yet initiate conversations with others, even with native speakers. They also cannot understand many words and grammatical structures that are used by more proficient speakers, so social interaction is more difficult for them, even for the more advanced ones.

Our results on the role of LLS in English language achievement and attitude confirmed the statistically significant effect of LLS on background variables. English language achievement was directly influenced by the metacognitive and cognitive fields and attitudes and indirectly affected by the meta-affective and affective fields, as well as the metasocial and social fields. Our model could not confirm any direct or indirect effect of the metamotivational and motivational fields on attitude or English language achievement. This may be because motivational components form distinct factors and their role differs somewhat in predicting language achievement. These results are in line with previous findings (Platsidou and Kantaridou, 2014; Habók and Magyar, 2018a), which also concluded the outstanding role of attitudes, which is an important predictor of language achievement and reinforces the role of strategy use. In summary, strategy use influences English language achievement through attitude to language learning in a statistically significant way.

## Conclusion

The main objective of the study was to find evidence for the role of strategy use in students’ achievement at the beginner/elementary level of English language learning. As a result, the strategy use preferences of the sample differed somewhat from the findings of previous research, as the affective and motivational fields were the ones the students preferred the most. This may be due to the fact that young children are more likely to use strategies that are rather emotional and related to their personality traits than strategies that require deeper understanding, specific learning techniques, and awareness, such as cognitive strategies. The use of social strategies was also quite low, probably owing to the low level of foreign language communication skills in the sample. As regards the different proficiency levels, more frequent strategy use was observed among the more proficient learners, a statistically significant finding. However, the patterns of strategy use were almost the same across the groups. The only difference was that the more proficient learners mostly preferred the affective field, while the less proficient ones mostly employed motivational strategies. This indicates that students at a higher level have more confidence to speak up and show how they feel about learning English. Learners with lower proficiency at this age often try to show that they are motivated, that is, that they are trying and want to achieve good results and present a good image of their own performance. The study also highlighted the importance of attitude; from the results, it can be concluded that, even at the beginner/elementary level, strategy use can affect language achievement and that a student’s attitude is an important predictor and plays an important role as mediator between strategies and language achievement. This can have a positive impact on classroom performance and highlights the importance of teaching students about learning strategies.

## Limitations

There are some limitations to consider in the study. First, the questionnaire was administered to fifth-grade students, who were at the beginner/elementary level of their English language learning. Thus, generalizability cannot be confirmed, and more research is needed across higher grades and higher proficiency learners. Second, we had difficulty identifying the affective domain in the first version of the questionnaire. For the fields in the present measurement tool, we have succeeded in identifying the affective and meta-affective domains of LLS. However, they still have to be optimized. Additional research is also called for with regard to the motivational components. Third, other underlying factors should be included in the investigation, such as self-efficacy, self-esteem, and self-concept.

## Pedagogical Implications

The study points out that the role of learning strategies is substantial for the students in their language learning. Learning English is a complex process for Hungarian fifth graders. English pronunciation, vocabulary, and grammar are very different from those of Hungarian. For these learners, grammatical rules are often abstract phenomena, and it is difficult for them to associate meaning with the words they say and write. Furthermore, reading and listening comprehension are also influenced by many factors. The results draw attention to the paramount importance of teaching LLS, which can promote greater success among language learners. In addition, it is essential how consciously strategies are employed. Teachers are strongly urged to include strategy training in their courses. Strategy training can be conducted either in the form of an embedded sub-course in any of the subjects or in an independent form as an individual course. Strategy courses integrated into a school subject provide specific help for students learning that specific course material. For example, language learning strategies paid students in learning grammatical formulae or vocabulary in a foreign language, while general strategy courses help students to learn strategies that can be used in other school subjects, such as reading and writing strategies.

Another implication of the study is that motivation and attitude also influence language achievement in a statistically significant way. Creating a learner-friendly and encouraging atmosphere is therefore essential. The findings from our research have provided important insights into these issues for classroom practice.

## Data Availability Statement

The datasets presented in this article are not readily available because the datasets are confidential and cannot be shared with third parties. Requests to access the datasets should be directed to AH, habok@edpsy.u-szeged.hu.

## Ethics Statement

The studies involving human participants were reviewed and approved by IRB at the Doctoral School of Education, University of Szeged. Written informed consent to participate in this study was provided by the participants’ legal guardian/next of kin.

## Author Contributions

AH and AM designed the study and implemented the data collection, as well as analyzing the data and participating in completing the manuscript. GM supervised the research and provided support. All the authors contributed to the editing and revision of the study and approved the final version of the manuscript.

## Conflict of Interest

The authors declare that the research was conducted in the absence of any commercial or financial relationships that could be construed as a potential conflict of interest.

## Publisher’s Note

All claims expressed in this article are solely those of the authors and do not necessarily represent those of their affiliated organizations, or those of the publisher, the editors and the reviewers. Any product that may be evaluated in this article, or claim that may be made by its manufacturer, is not guaranteed or endorsed by the publisher.

## Funding

This research was supported by the Research Programme for Public Education Development, Hungarian Academy of Sciences (grant KOZOKT2021-16) and a Hungarian National Research, Development and Innovation Fund grant (under the OTKA K135727 funding scheme).

## Appendix

**Table:** Revised Version of the Self-Regulated Foreign Language Learning Strategy Questionnaire (SRFLLSQ).

When I learn English, …	
Metacognitive	
1.	I think of the relationships between what I already know and new things I learn in English.
2.	I first skim an English passage, then go back and read carefully.
3.	I look for opportunities to read as much as possible in English.
4.	I write notes, messages, letters or reports in English.
5.	I plan my schedule so I will have enough time to study English.
6.	I pay attention when someone is speaking English.
7.	I make summaries of information that I hear or read in English.
8.	I try to find out how to be a better learner of English.
Cognitive	
9.	I connect the sound of a new English word and an image or picture of the word to help me remember the word.
10.	I use the English words I know in different ways.
11.	I find the meaning of an English word by dividing it into parts that I understand.
12.	I use new English words in a sentence so I can remember them.
13.	I try to find patterns (grammar) in English.
14.	I try not to translate word for word.
Meta-affective	
15.	I notice if I am tense or nervous when I am studying or using English.
16.	I encourage myself as I learn English so that I can learn what I would like.
17.	I read in English as a leisure-time activity.
18.	I organize my English language learning so that I always enjoy doing it.
19.	I plan my English language learning so that I can perform better.
20.	I have more success learning English when I feel like doing it.
21.	I talk to others about how I feel when learning English.
22.	I give myself a reward or treat when I do well in English.
Affective	
23.	I try to relax whenever I feel afraid of using English.
24.	I encourage myself to speak English even when I feel afraid of making a mistake.
25.	I try to overcome my anxiety if learning English is difficult.
26.	Making mistakes does not take away my desire to use English language.
27.	It gives me a good feeling when I do well in English.
28.	I am good at learning English.
29.	I like learning English.
30.	I am happy when I can use my knowledge of English in other school subjects.
Metasocial	
31.	I try to learn about English-language cultures and/or other cultures through English.
32.	I look for people I can talk to in English.
33.	I look at English-language TV shows, movies or websites to get to know the cultures of English native speakers and/or other cultures through English.
34.	I choose leisure activities where I encounter English-language cultures and/or other cultures through English as well.
35.	I plan what I want to find out about the cultures of English speakers and/or other cultures through English.
36.	I practise English with my peers.
37.	I look for similarities and differences between my own culture and the cultures of English native speakers and/or other cultures through English.
38.	Getting to know English-language cultures helps me to learn the language.
Social	
39.	I start conversations in English.
40.	I make up new words in English if I do not know the right ones.
41.	When I speak with highly proficient speakers of English, I think it is important to get acquainted with their culture.
42.	The more I learn about English-language culture(s) and/or other cultures through English, the more I love English.
43.	I try to discover similarities and differences between my own culture and English-language cultures and/or other cultures I’m learning about through English.
44.	I like talking to other students about English-language cultures and/or other cultures I’m learning about through English.
Metamotivational	
45.	I pay attention to the types of English learning tasks that make me excited.
46.	I plan ahead for what I’m going to learn in English in a week or two.
47.	I add something motivating to my English learning environment, such as pleasant music.
48.	I pay attention to making it interesting to learn English.
Motivational	
49.	I give myself a reward for good progress or achievement.
50.	I use positive self-talk about my reasons for achieving my aims.
51.	I regard learning English as a game.
52.	I believe that I’m fully capable of doing English learning tasks.
